# Computerized data treatment for an HPLC-GFAAS system for the identification and quantification of trace element compounds

**DOI:** 10.1155/S1463924693000082

**Published:** 1993

**Authors:** G. Kölbl, K. Kalcher, K. J. Irgolic

**Affiliations:** Institut für Analytische Chemie Karl-Franzens Universität Universitätsplatz 1 Graz A-8010 Austria

## Abstract

Liquid chromatographs, coupled with graphite furnace atomic absorption spectrometers, have been widely used for the identification and quantification of trace element compounds. The quantification of the discontinuous signals from the spectrometer defining a chromatographic band is very much a matter of judgement and therefore prone to error. This paper describes a system which links a high-performance liquid chromatograph via a ‘Brinckman’ flowthrough cup to a Hitachi Zeeman graphite furnace atomic absorption spectrometer equipped with an autosampler. The introduction of aliquots from the column effluent and the analysis sequence is computer-controlled through a home-built interface. The signals from the spectrometer are passed through an analoguedigital converter and processed by selectable algorithms. The software offers a variety of options for processing the chromatographic data, such as data smoothing, Gaussian or spline interpolation, and trapezium or Simpson integration.

This system was used to separate and determine selenite and selenate in aqueous solution with absolute detection limits (3 σ) of 23 ng Se for selenite and 16 ng Se for selenate. This system can be adapted to other spectrometers, provided that the required connections to the electronics can be made.


Journal of Automatic Chemistry, Vol. 15, No. 2 (March-April 1993), pp. 37-45

### Computerized data treatment for an HPLC-GFAAS system for the identification and quantification of trace element compounds

**G. Kölbl, K. Kalcher and K. J. Irgolic**

*Institut für Analytische Chemie, Karl-Franzens Universität, Universitätsplatz 1, A-8010 Graz, Austria*

*Liquid chromatographs, coupled with graphite furnace atomic absorption spectrometers, have been widely used for the identification and quantification of trace element compounds. The quantification of the discontinuous signals from the spectrometer defining a chromatographic band is very much a matter of judgement and therefore prone to error. This paper describes a system which links a high-performance liquid chromatograph via a 'Brinckman' flow-through cup to a Hitachi Zeeman graphite furnace atomic absorption spectrometer equipped with an autosampler. The introduction of aliquots from the column effluent and the analysis sequence is computer-controlled through a home-built interface. The signals from the spectrometer are passed through an analogue-digital converter and processed by selectable algorithms. The software offers a variety of options for processing the chromatographic data, such as data smoothing, Gaussian or spline interpolation, and trapezium or Simpson integration.*

*This system was used to separate and determine selenite and selenate in aqueous solution with absolute detection limits (3 σ) of 23 ng Se for selenite and 16 ng Se for selenate. This system can be adapted to other spectrometers, provided that the required connections to the electronics can be made.*

### Introduction

In the past, analytical research sought to lower the detection limits for existing methods and to invent new techniques for total element determinations. Research in recent years, however, has focused on the identification and quantification of trace element compounds. This research area, which is often called 'speciation' [1], usually employs chromatography for the separation of trace element compounds and another suitable instrumental method for their identification and quantification.

Organic compounds containing metals or metalloids, such as cobalt, tin, lead, mercury, selenium and arsenic, occur at low concentrations in environmental samples (water, air, soil, plant, animal and human tissues). These compounds differ in their physical and chemical properties: some are volatile, neutral molecules ($Me_4Pb$, $Me_2Se$, $Me_3As$), some are involatile and carry a charge ($Me_3AsCH_2CH_2OH^+$, $Me_3Se^+$); and others are co-ordination compounds of low or high molecular mass with widely different stabilities. For the separation of volatile compounds and of compounds that can be converted into volatile derivatives, gas chromatography can be used. However, most trace element compounds are not volatile and cannot be transformed into such species without loss of information about their chemical nature. Such substances are best separated with high performance liquid chromatography.

Environmental samples contain, in addition to the species of interest, many other compounds, often at much higher concentrations than the analytes to be determined. Detectors that exploit physico-chemical changes (refractive index, light absorption, conductivity) of the column effluent to detect analytes are neither element- nor molecule-specific. Detectors specific to an element can considerably simplify the chromatography, because only the compounds containing this element need to be separated. Even large excesses of co-eluting components do not usually interfere with the analysis. Such detectors for liquid chromatography are spectrometers that respond to atomic transitions.

Electrothermal atomic absorption spectrometers operate element-specifically and offer low detection limits. However, the sample (generally a solution) must be dried and ashed prior to atomization. These sequential steps prevent the direct coupling of a continuously working chromatograph with the graphite furnace atomic absorption spectrometer: several systems have been developed to overcome this difficulty [2-9] and these methods were recently summarized [10 and 11]. Brinckman and co-workers [3] developed a system based on autosamplers: the effluent is routed through a flow-through cup from which the autosampler periodically transfers an aliquot into the furnace. The Brinckman system produces a series of atomic absorption signals that define a chromatographic band for each analyte containing the element to be detected. Such a series of signals can provide quantitative information about the analyte by summing the intensities of the signals belonging to a band, or by measuring the area under the curve obtained by connecting the signal maxima in sequence [15]. However, these procedures are cumbersome and associated with considerable error. Although several automated HPLC-GFAAS systems have been developed [2-9], the data collection and treatment for these systems has not yet been automated.

A Brinckman system is described in this paper. The introduction of the aliquots from the eluent and the analysis sequence is computer-controlled via a home-built electronic interface. The signals from the spectrometer are passed through an analogue-digital converter and processed by selectable algorithms that produce qualitative and quantitative information about the analytes. The results can be displayed on screen or printed out.

---

Correspondence should be addressed to Dr. Irgolic.
The software package described here including all source codes, can be obtained from the authors; send requests to Dr. Irgolic together with a formatted floppy disk ($5\frac{1}{4}$ in or $3\frac{1}{2}$ in).

0142-0453/93 $10.00 © 1993 Taylor & Francis Ltd.

### Instrumentation

The chromatographic system consisted of a double-headed pump (Waters 600 E Multisolvent Delivery System), an injector (Waters U6K Injector), a pre-column (Hamilton), and an analytical column mounted in a thermostated chamber (Waters TCM Temperature Control System). The effluent from the column passes through a flow-through cell made of polytetrafluoroethylene (Teflon) with a dead volume of 0·03 ml [3]. The liquid enters the cell through a dead volume connector at the bottom of the cell. Excess effluent, not used for injections into the graphite tube, is removed at the top of the cell by a water aspirator [3]. The atomic absorption spectrometer system consisted of a Zeeman atomic absorption spectrometer (Hitachi Model 170-70) and an autosampler (Hitachi 170-0125).

The analysis sequence was controlled and the data treated with a personal computer (IBM AT-386 compatible, 33 MHz), equipped with 4 MB RAM, VGA graphics card (1 MB, 1024 × 768 pixels), a monitor, a mathematical co-processor, 120 MB hard disk, and an analogue-to-digital/digital-to-analogue converter (ADDA converter RTI 815 from Analog Devices).

### *Description of the system*

The graphite furnace atomic absorption spectrometer operates discontinuously. An analysis cycle, which takes 2 min, consists of transfer of an aliquot from the flow-through cell to the furnace by the autosampler; drying, ashing, and atomizing the sample; and cooling the graphite furnace to room temperature [12]. This relatively long period between two consecutive determinations needs to be shorter – otherwise chromatographic bands may not be well defined in the chromatogram and narrow bands may be missed. Therefore, a system was designed to independently control the autosampler and the furnace. This interface was inserted between the control module for the autosampler and the power unit for the graphite furnace (see figure 1).

The wiring diagram for the interface is shown in figure 2; pin connections and assignments are listed in table 1. All the connecting lines between the controller for the autosampler (A/S) and the power unit for the furnace (P/S) were disconnected and reconnected as shown in figure 2. The interface has two main tasks: initiation of actions, and supervision of the active state of autosampler and spectrometer. Inverse TTL (transistor-transistor logic) signals of +5 V are provided by the spectrometer (pin No. 5 of P/S) and by the autosampler (pin No. 18 of A/S), indicating whether the units are active or inactive. The interface changes the +5 V signals to −5 V signals, which are recognized by the digital port of the ADDA converter (this operation could also be accomplished with an operational amplifier). The signals are then transmitted from the ADDA converter to the computer. For activating or deactivating the autosampler, pins No. 1 and No. 2 of the A/S are briefly short-circuited at a time selectable by the operator. Similarly, connection of pin

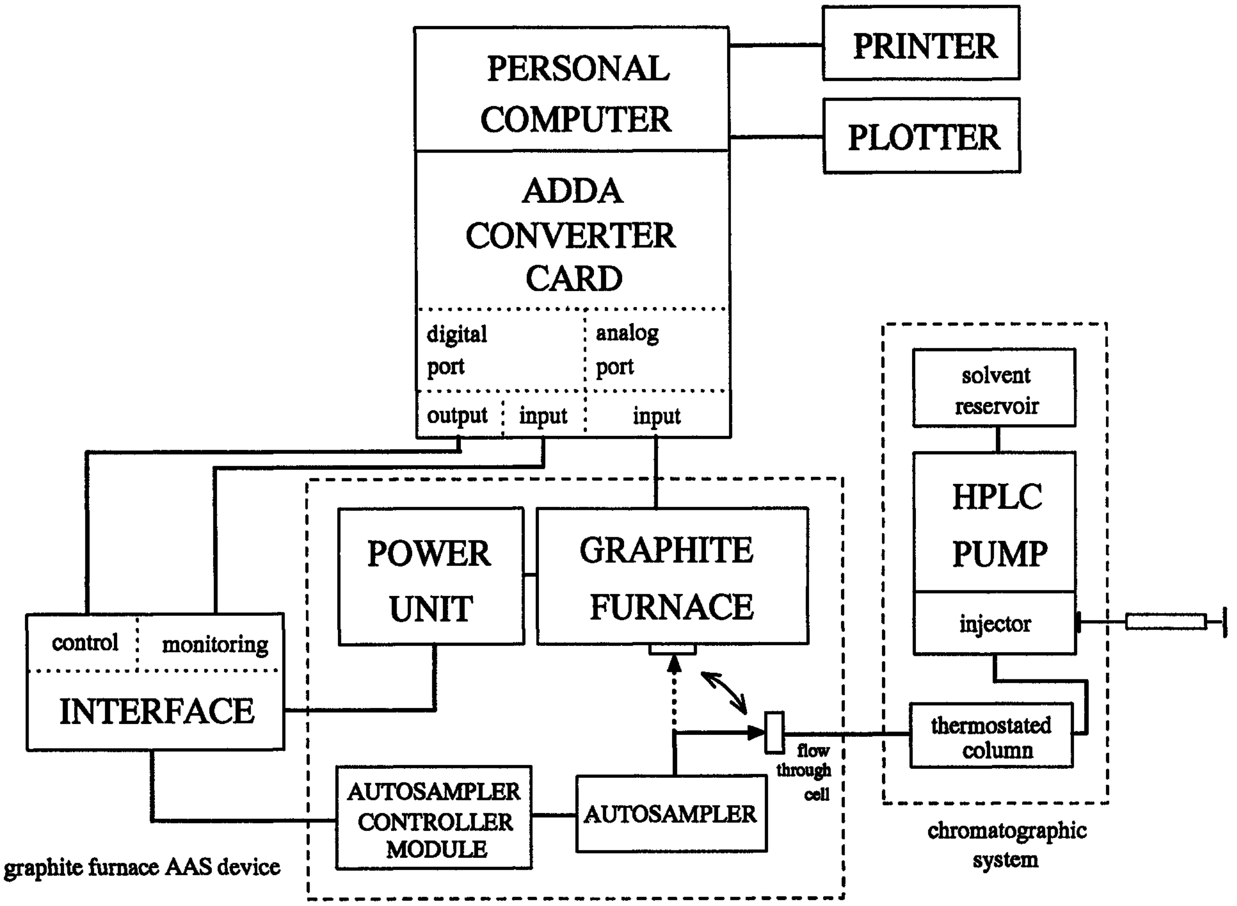

*Figure 1. Schematic diagram showing the HPLC-GFAAS system described.*

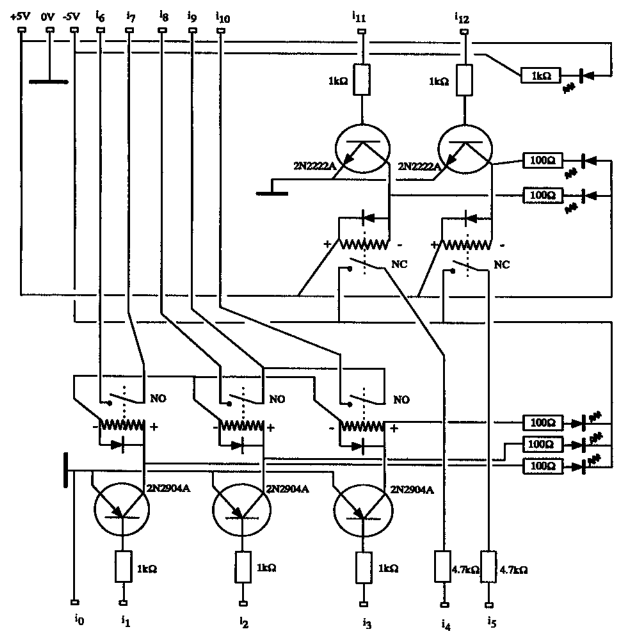

*Figure 2. Wiring of the interface which controls the GFAA spectrometer via a personal computer (for pin assignments see table 1).*

*Table 1. Pin assignments for the interface.*

| Device | Pin number | Assignment | Connected to pin number of the interface |
|---|---|---|---|
| Autosampler | 1 | Start/stop A/S | $i_6$ |
| Autosampler | 2 | Start/stop A/S | $i_7$ |
| Autosampler | 18 | Monitor A/S | $i_{12}$ |
| Power unit | 13 | Stop P/S | $i_8$ |
| Power unit | 11 | Ground | $i_9$ |
| Power unit | 16 | Start P/S | $i_{10}$ |
| Power unit | 5 | Monitor P/S | $i_{11}$ |
| ADDA converter | 1 | Ground | $i_0$ |
| ADDA converter | 3 | Digital output bit No. 0 | $i_1$ |
| ADDA converter | 7 | Digital output bit No. 2 | $i_2$ |
| ADDA converter | 9 | Digital output bit No. 3 | $i_3$ |
| ADDA converter | 4 | Digital input bit No. 0 | $i_4$ |
| ADDA converter | 6 | Digital input bit No. 1 | $i_5$ |

No. 11 with pin No. 16 of the power supply initiates the sequence drying-ashing-atomization. Bridging pins No. 11 and No. 13 of the P/S stop the action of the spectrometer. These short-circuits are effected by relays which are controlled with a TTL-compatible signal (−5 V) from the computer. All the TTL signals are generated or monitored by the ADDA converter.

To enable data manipulation with the computer, the recorder output of absorption and background signals of the AAS is analogue-to-digital converted via the analogue port of the ADDA converter. The software system (called 'AAS-IFKK') was developed to control the progress of the analysis via the interface, and to monitor and manipulate the data.

### Data handling and evaluation

The computer program for this system is written in QBASIC 4.0 (MS Basic Compiler Version 6.0); the actions of the ADDA converter are accomplished with factory-supplied driver routines. The program performs the following tasks:

(1) Control of the auto-sampler by restarting it in a repetitive manner with selectable idle periods.
(2) Monitoring of the absorption and background signals during drying, ashing, and atomization.
(3) Evaluation of the chromatogram and of chromatographic parameters.
(4) Graphic and numeric representation of data (printer, plotter) and archiving of data.

The flow chart for the program is shown in figure 3. All inputs and selections of parameters are accomplished via menus. The main menu allows the specific task (data collection or manipulation of previously recorded data) to be selected.

The HPLC-GFAAS system is controlled via the digital port of the ADDA converter. Relays in the interface are activated by producing a TTL signal of −5 V on the digital output port encoded as a bit pattern. The values set manually at the control unit of the spectrometer for the duration for drying, ashing, and atomization are taken into the program by performing an analysis without starting the chromatographic system ('test cycle'). The program will store and recall these parameters. The idle period of the A/S is adjusted to allow the furnace to cool sufficiently to accept the next sample without sputtering. After the atomization of selenium at 2600 °C, 10s were found to be sufficient. The entire detection system may then be started with the program at the same time as the chromatographic system is switched on manually.

### *Evaluation of one data point of the chromatogram*

Table 2 lists, as an example, the part of the program that performs the control task and the data handling between the atomic absorption device and the computer. A periodic cycle for obtaining one data point of the chromatogram begins with activating the autosampler by setting the appropriate bit at the ADDA converter (digital output bit No. 0) to −5 V, prompting the relay of the interface to short-circuit pin No. 1 and pin No. 2 at the autosampler (A/S). Monitoring the autosampler condition ('on' or 'off') at pin No. 18 (digital input bit No. 1) provides a check on whether or not the autosampler had finished its action. The power supply receives the signal from the digital output bit No. 3 via the corresponding relays (pin Nos. 11 and 16 at the power unit) to start the drying, ashing, and atomizing. The data that are read from the analogue port of the converter (absorption signal) with a preselected conversion rate (1 to 26 points/s) are displayed on the screen. The atomization-state signal (pin No. 5 at the power unit), which is monitored at the same time (digital input bit No. 0), indicates whether

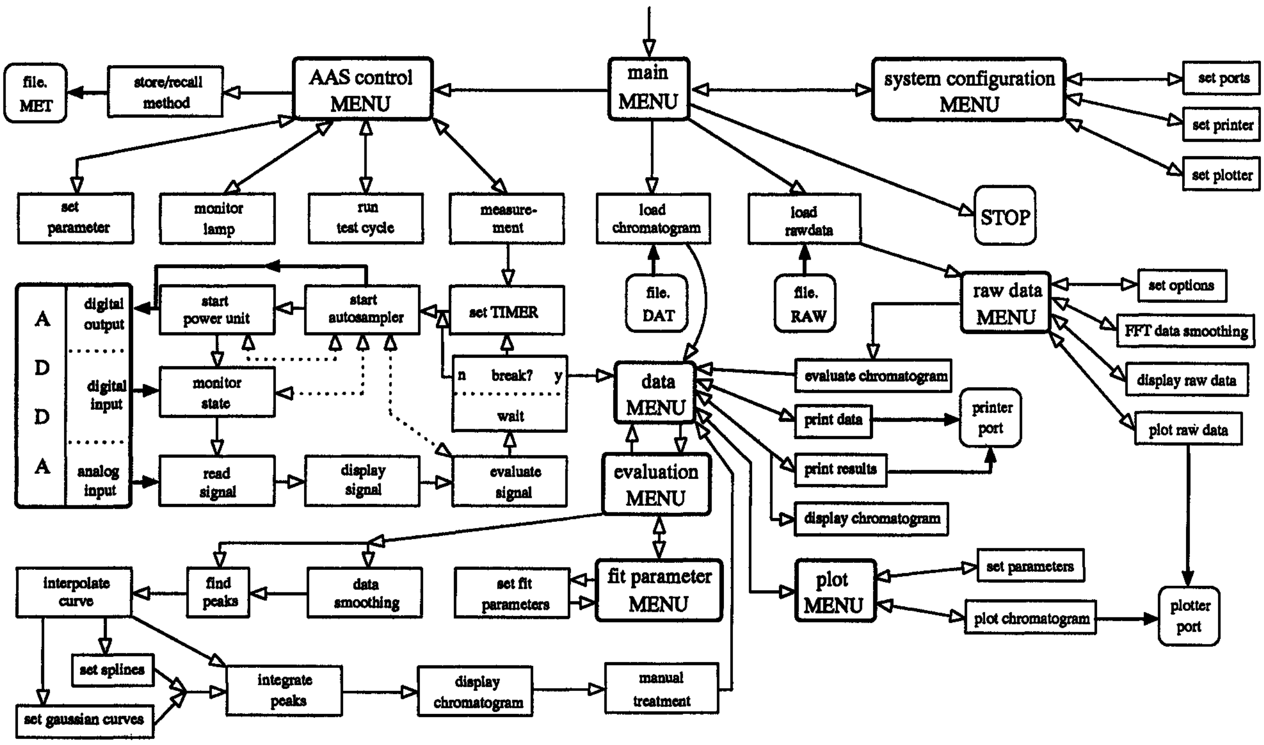

*Figure 3. Flow chart of the control program for the GFAA spectrometer, autosampler, and data treatment.*

absorption is measured during drying or ashing, or during atomization. If the raw data are to be saved (an option available in the AAS control menu), all the data points are stored in a file. From these raw data, the absorption is evaluated; a single value for the chromatogram is then provided. The algorithm is straightforward: for a selected period during drying/ashing the mean value of the absorption is calculated and taken as a base-line for the atomization signal. Evaluation of the absorption during atomization can be performed in two ways by choosing a corresponding option: this can be either the maximum signal, or the area between the signals and the base-line over the whole period. To reduce the noise in the base-line and signal, the program offers the possibility of Fourier-transform data smoothing using the algorithm of Press *et al.* [13]. The time needed for one analysis cycle depends on the parameters chosen. Atomizations can be performed at intervals as short as 30 s, because the autosampler can be restarted when drying/ashing/atomization is still active.

This repetitive registration of absorptions for the chromatogram is stopped either manually via the computer by pressing the ESC button, or when the time limit set for the analysis is exceeded.

### *Manipulation of the chromatographic data*

The subsystem of the program for the treatment of chromatographic data may be entered directly after measurement of the absorption signals or after loading previously recorded raw data or chromatographic data. Such raw chromatograms can be evaluated by options which can be chosen via a menu.

#### *Data smoothing*

If desired, the chromatographic data can be smoothed by Fourier transformation. If only a relatively small number of data is available (which is the case when using this kind of analytical device), smoothing cuts the peak maxima significantly and so should be avoided.

#### *Base-line evaluation*

The base-line of the chromatogram is set either manually or automatically (default). In the automatic mode, the lowest value of absorption in the chromatogram is taken, and a mean value of all other data lying within a pre-defined range ('noise' parameter, given as a percentage of the $y$-axis) is calculated as the base-line.

#### *Finding of peaks*

After setting the base-line, a search routine detects chromatographic peaks. This routine was designed to take account of the experimentally caused noise in chromatograms.

The program calculates the difference in the heights of two consecutive signals, starting with the first signal in the chromatogram. Differences with opposite signs are summed separately to produce values for the variables 'DIFFPOS' and 'DIFFNEG'. If two consecutive differences change signs, then the preceding summation variable is examined to discover whether it is larger or smaller than the pre-defined noise-parameter 'NOISE'. If the variable is smaller, then it is set to zero. Thus, if a negative difference is encountered after a sequence of positive ones, DIFFPOS is set to zero, if it is smaller than NOISE.

*Table 2. Part of the program AAS-IFKK which controls the interface.*

```
RunMethod:
   rate! = 1! / (digitrate% * 1!)
   f! = 1! / 4096!: IF gain% = 500 THEN f! = f! * 10
   ltt% = 4: las% = 10:  lda% = 13: lat% = 16: lb% = 19: lw% = 22: cl% = 66
   IF gain% = 10 THEN
      ypmax = 1!: y$ = "Volt": yy$ = y$
      ELSE
      ypmax = 10!: y$ = "milliVolt": yy$ = y$
   END IF
   ypmin = 0: xpmin = 0: xpmax = drytime! + ashtime! + atomtime! + btime!
   x$ = "seconds"
   GOSUB GraphicScreen
   LINE (xpmax, ypmin)-(xpmax, ypmax), 15
   LINE (xpmax, ypmax)-(xpmin, ypmax), 15
   IF basetime! > 0! THEN LINE (basetime!, ypmin)-(basetime!, ypmax), 10
   LINE (datime!, ypmin)-(datime!, ypmax), 11
   LINE (datime! + atomtime!, ypmin)-(datime! + atomtime!, ypmax), 11
   IF ifinit% = 0 THEN CALL Initialize(errr%): ifinit% = 1
   LOCATE sl%, 5: PRINT "Press any key to start measurement [ESC to skip]";
   SHELL "aasvm aasstm.spr pl int xt 0"
   cx$ = "": WHILE cx$ = "": cx$ = INKEY$: WEND
   IF cx$ = CHR$(27) THEN SCREEN 0: RETURN
   LOCATE sl%, 5: PRINT SPACE$(74);
   LOCATE sl%, 7: PRINT "active cycle:"
   LOCATE ltt%, cl%: PRINT "total time:"
   cycle% = 0: ifas% = 0
   COLOR 14: LOCATE las%, cl%: PRINT "A/S:"
   COLOR 13: LOCATE lda%, cl%: PRINT "dry/ash:"
   COLOR 12: LOCATE lat%, cl%: PRINT "atomize:"
   COLOR 11: LOCATE lb%, cl%: PRINT "booster:"
   IF waittime! > 0! THEN
      COLOR 10: LOCATE lw%, cl%: PRINT "wait:"
   END IF
   wtime = datime! + atomtime! + waittime!
   IF wtime <= datime! THEN
      ifstartas% = 3
      ELSEIF wtime <= datime! + atomtime! THEN
      ifstartas% = 2
      ELSEIF wtime <= datime! + atomtime! + 3! THEN
      ifstartas% = 1
      ELSE
      ifstartas% = 0
   END IF
   IF ifraw% <> 0 THEN
      IF ifrawopen% = 1 THEN CLOSE #200
      OPEN raw$ FOR OUTPUT AS #200: ifrawopen% = 1
      filelu% = 200: GOSUB WriteHeader
   END IF

   start! = TIMER

   ifende% = 0: ifpson% = 0

   DO

      cycle% = cycle% + 1: LOCATE sl%, 21: PRINT USING "####"; cycle%

      LOCATE sl%, 30: PRINT SPACE$(30)

      IF ifende% = 1 THEN LOCATE sl%, 2: PRINT "last"

'==== start A/S

      COLOR 14
      IF ifas% = 0 THEN ifas% = 1: GOSUB AutoSamplerOn
      ifas% = 1
      IF INKEY$ = CHR$(27) THEN GOSUB BreakCycle
      DO
         CALL dinb(card%, port%, monitoras%, value%, errr%)
         LOCATE las% + 1, cl%: PRINT USING "#####.##"; TIMER - startAS!
         LOCATE ltt% + 1, cl%: PRINT USING "#####.##"; TIMER - start!
         IF INKEY$ = CHR$(27) THEN GOSUB BreakCycle
      LOOP UNTIL value% = 1
      GOSUB AutoSamplerOff
      LOCATE las% + 1, cl%: PRINT SPACE$(8)

'==== dry/ash

      COLOR 13
      CALL dot(card%, port%, pson%, errr%)
      sc! = TIMER
      WHILE (TIMER - sc!) < st: WEND
      CALL dot(card%, port%, value0%, errr%)
```

*Table 2 (continued)*

```

      ifpson% = 1: startC! = TIMER: stC! = startC!

      CALL ain(card%, chan1%, gain%, vall1%, errr%)

      CALL ain(card%, chan2%, gain%, vall1b%, errr%)

      sig(1) = (vall1% + 2048) * f!

      b(1) = (vall1b% + 2048) * f!

      t(1) = 0

      i% = 1: ifstart% = 0

      IF ifstartas% = 3 THEN ifstart% = 1

      v% = 0: ll% = lda% + 1: GOSUB GetData: drysignal% = i%

'==== atomize

      COLOR 12
      ifstart% = 0: IF ifstartas% = 2 THEN ifstart% = 1
      stC! = TIMER: v% = 1: ll% = lat% + 1: GOSUB GetData: atomsignal% = i%

'==== booster

      COLOR 11: ll% = lb% + 1

      startw! = TIMER: startC1! = startw!: stC! = startC1!

      WHILE (TIMER - startw!) < btime!

         GOSUB NewValue

         IF ifstartas% = 1 AND ifende% = 0 AND ifas% = 0 THEN

            IF startC2! - startw! + datime! + atomtime! > wtime THEN

               ifas% = -1: GOSUB AutoSamplerOn

            END IF

         END IF

      WEND

      GOSUB PowerSupplyOff

      LOCATE lb% + 1, cl%: PRINT SPACE$(8)

      LOCATE sl%, 60: PRINT SPACE$(19);

'==== wait

      COLOR 10

      GOSUB ClearCurve

      sssC! = startC! - start!: GOSUB EvaluateSignal

      IF ifende% = 1 AND ifas% = 0 THEN EXIT DO

      IF waittime! > 0! THEN

         WHILE TIMER - startw! < waittime!

            LOCATE lw% + 1, cl%: PRINT USING "#####.##"; TIMER - startw!

            LOCATE ltt% + 1, cl%: PRINT USING "#####.##"; TIMER - start!

            IF INKEY$ = CHR$(27) AND ifas% = 0 THEN GOSUB BreakCycle: EXIT DO

         WEND

         LOCATE lw% + 1, cl%: PRINT SPACE$(8)

      END IF

      IF INKEY$ <> "" THEN EXIT DO

   LOOP UNTIL (ifende% = 1 AND ifas% = 0) OR (TIMER - start!) / 60! > maxtime!

   FOR ii% = ltt% TO 23

      LOCATE ii%, cl%: PRINT SPACE$(11)

   NEXT ii%

   LOCATE sl% + 2, 30: COLOR 14: PRINT "*** END OF MEASUREMENT ***"

   IF ifas% <> 0 THEN GOSUB AutoSamplerOff

   IF ifrawopen% = 1 THEN CLOSE #200: ifrawopen% = 0

   GOSUB MenuScreen

   IF RIGHT$(type$, 1) = " " THEN yy$ = yy$ + "*s"

   datum$ = DATE$: hour$ = TIME$

RETURN

```

The treatment is applied to DIFFNEG if a negative-positive sequence of differences is detected. If DIFFPOS is larger than NOISE, a peak is found. All signals in the chromatogram are processed in this way. A peak maximum is located when the absolute value of DIFFNEG becomes larger than NOISE; DIFFPOS is then set to zero. The highest signal within this range is taken as peak maximum. Figure 4 illustrates the process.

$D_i$ in figure 4 corresponds to the difference of the heights $H_{i+1} - H_i$ of the signals $S_{i+1}$ and $S_i$. First $D_1$ ($H_2 - H_1$), a positive difference, is assigned to the variable DIFFPOS. $D_2$ ($H_3 - H_2$) is negative and assigned to DIFFNEG. DIFFPOS is set to zero, because it is smaller than NOISE. $D_3$ ($H_4 - H_3$) is positive again and prompts DIFFNEG to become zero. $D_4$ ($H_5 - H_4$) is positive and added to DIFFPOS. Because DIFFPOS is now larger than NOISE, a peak must be present. $D_5$ ($H_6 - H_5$) is

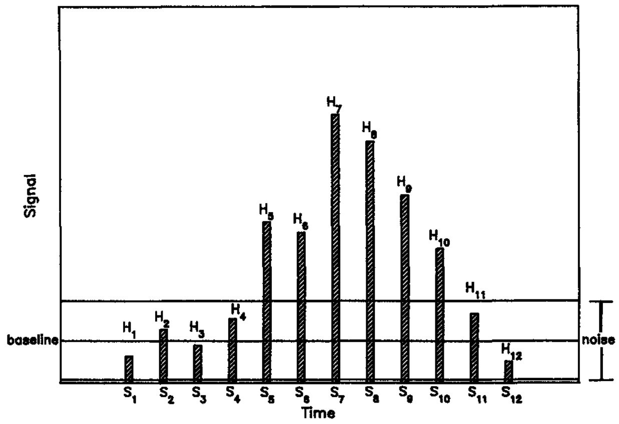

*Figure 4. Example of the procedure for finding peaks.*

negative and added to DIFFNEG. DIFFPOS ($D_4$) is not changed, because it is larger than NOISE and DIFFNEG is smaller than NOISE. $D_6$ is positive again and added to DIFFPOS, prompting DIFFNEG, which is smaller than NOISE, to be set to zero. $D_7$ and $D_8$ are summed up to DIFFNEG, which is now larger than NOISE; thus $H_7$, as the highest signal in this range, is taken as the peak maximum, and DIFFPOS ($D_4 + D_6$) is set to zero.

Three parameters characterize each peak: maximum, left limit, and right limit. The limits are found by determining, starting from the maximum in direction of either higher or lower retention times, the point on the retention time axis, at which the curve falls under the base-line plus half of the noise. The example in figure 4 shows $S_9$ as the left limit and $S_{11}$ as the right limit. If there is no such a peak limit found between two consecutive peaks (poorly resolved peaks), the lowest signal between the maxima is taken as the corresponding peak limit.

*Interpolation*

The type of interpolation that may be applied to the raw data of the chromatogram strongly depends on the appearance of the chromatogram. Therefore, some options are provided for refining the chromatogram: no interpolation, interpolation with splines, or interpolation with Gaussian curves. Splines are applicable if the noise in the raw chromatogram is very low; otherwise, as can be expected, oscillations occur. For calculating splines the algorithm of Press *et al.* was used [14].

Interpolations with Gaussian curves are performed in a straightforward manner. A Gaussian curve is positioned at every peak, and its parameters are then iterated until the sum of the squares of the deviations of the experimental data from the curve (calculated data) is at a minimum. To model unsymmetrical peak-shapes and tailings of peaks, the peak cannot be represented by a simple Gaussian curve, but, rather, must be modelled with two halves of different curves representing the left and the right part of the peak.

Therefore, four parameters characterize the Gaussian shape: position of the maximum, height of the maximum, left width, and right width. The initial Gaussian curves are established with the maximum and height representing the values generated by the peak-finding procedure; the widths of the left or right half of the curve, $w_L$ and $w_R$, are actually estimated as a third of the distance, $x_L$ or $x_R$, between the peak maximum $t_{max}$ and the left or the right limit, $t_L$ or $t_R$ (see figure 5). Thus, one half of the initial Gaussian curve is approximated by a triangle with two rectangular sides, h and $x_L$ or $x_R$. Overlapping peaks are represented by overlapping triangles. All four parameters, $w_R$, $w_L$, h, and $t_{max}$, are then iterated until the error reaches a minimum. Each parameter is initially changed by +2%, and the error is recalculated. If the error decreases, the program proceeds to change the parameter again by +2%; if the error increases, the parameter is altered by half of the previous change with reversed sign. Repetitive iterations are performed until the error is at a mimimum. The parameters are optimized in the sequence: widths, height, position. Because the parameters are dependent on each other, the resulting values are iterated again until a mimimum of error is detected by the program. The optimized Gaussian curve is added to the final chromatogram. All peaks are processed in the same manner. Finally, the peak limits are redetermined with the synthesized curve.

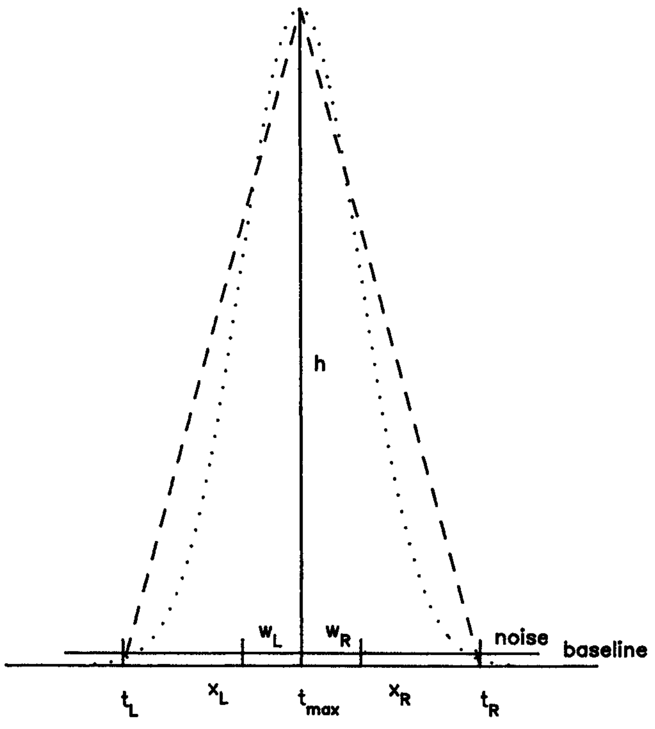

*Figure 5. Peak parameters for the interpolation of Gaussian curves.*

*Integration*

Peaks are integrated by either summing the signals between the peak limits (which is sometimes a good approach for equidistant data [15]), or by calculation of areas following trapezium or Simpson summation.

*Manual processing*

The processed curve is displayed on the screen and allows some additional manual manipulations. The curve can be tracked by cursors, offering the facilities of changing peak limits for integration, or elimination of minor peaks. If the limits are changed, the corresponding peak is reintegrated. The final results can be documented on a printer, a plotter or on files.

*Program characteristics*

The program is written in QBASIC 4.0. It consists of one main module and five sub-modules. The main module contains 2500 lines.

All modules were compiled in MicroSoft Basic Compiler Version 6.0 and linked with MS-Link Version 5.01.20. The program needs 246 kB memory.

Any instrument which is able to interpret the Hewlett-Packard graphic language can be used as the plotter.

### Application to the determination of selenite and selenate

To check the suitability of this coupled HPLC-GFAAS system for the determination of trace element compounds, selenite and selenate were quantified in aqueous solution. These compounds were separated on a strongly basic anion exchange column (ESA Anion III 250 × 4 mm, 10 μm) with an aqueous solution of potassium hydrogen phthalate (3 mM) as the mobile phase. The flow rate was 0·3 ml/min. Mobile phases were filtered through 0·2 μm cellulose nitrate filters prior to use.

GFAAS works discontinuously, so very narrow peaks in the chromatogram can be missed by the detector. To avoid this, low flow-rates and short intervals between individual analyses are necessary. The time required for one measurement was kept as short as possible by omitting two procedures normally part of determinations with GFAAS: ashing the sample, and cleaning the cuvette by heating. The optimal settings for the spectroscopic measurement were: drying 21 A (76 °C) for 30 s and atomizing 260 A (2600 °C) for 5 s. Injection of a sample into the graphite tube could be carried out as soon as the furnace had cooled to approximately 150 °C. The required time for cooling was evaluated with a thermocouple (Technoterm 9400, NiCr-Ni 0593/901) and was found to be at least 10 s after atomization. With these parameters the cycle time was 45 s.

Stock solutions were prepared by dissolving appropriate amounts of $Na_2SeO_3$ (Fluka 71950) and $Na_2SeO_4$ (Fluka 71947) in doubly distilled water to a final concentration of 1000 mg Se/l. Solutions of lower concentrations were prepared by dilution. For chromatographic analysis, 100 μl or 250 μl were injected onto the column with a microliter syringe (Hamilton). After setting the spectrometer, the chromatograph was started manually; the program was also started. Figure 6 shows the separation of selenite and selenate with this method.

Quantification of the obtained signals was performed in several ways: maximal signal of a peak, summation of signals within a peak [15], evaluation of peak cluster areas [15], or Gaussian curve interpolation with either trapezium or Simpson integration. The poor linearity of the calibration plot 'peak height of the most intense signal within a peak cluster' versus 'concentration' (see figure 7[d]) is the result of the discontinuous sampling of the column effluent. The possibility of an aliquot of the effluent always being taken exactly at the same position within a chromatographic band is small. Because aliquots are taken from slightly different positions within a band each time a chromatogram is produced, a linear calibration would simply be accidental and statistically unlikely. Signal summation, peak cluster area evaluation, and Gaussian curve interpolation calculate areas for chromatographic bands and smooth out signal variations caused by the discontinous sampling. The Gaussian algorithm was found to give the best results (see figure 7[a]); calibration curves were linear from 0·4 to 6 mg/l (250 μl sample volume) or

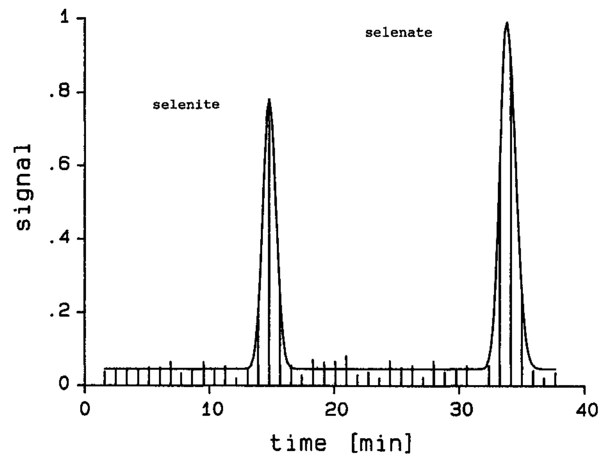

*Figure 6. Separation of selenite and selenate by the HPLC-GFAAS technique employing an anion exchange column: ESA Anion III 250 × 4 mm, 10 μm particles; mobile phase 3 mM potassium hydrogen phthalate adjusted to pH 7·4 with potassium hydroxide; flow rate 0·3 ml/min; 250 μl sample containing 1 μg selenium as selenite and 1 μg selenium as selenate; 20 μl of column effluent injected into the graphite furnace (DRY 21 A for 30 s, no ASH, ATOM 260 A for 5 s).*

from 1 to 10 mg/l (100 μl sample volume) for selenium as selenite or selenate.

The practical applicability of the proposed method was tested with two different samples. The first sample was an aqueous selenium standard solution ($SeO_2$ in dilute nitric acid, Merck 9915) and the second a solution, which is used to supply selenium to animals by subcutaneous or intramuscular injection. This solution contains selenium as selenite and several organic compounds in substantial excess to selenium. Both samples were diluted as required and injected onto the column without pretreatment. Quantification was accomplished with internal standard addition of selenite solutions. The chromatograms of both solutions showed that selenite was the only selenium compound present. The concentration of the Merck standard solution was found to be 1020 ± 60 mg Se/l (label 1000 mg/l); the solution used in veterinary medicine 480 ± 30 mg Se/l (label 500 mg/l). A standard solution certified for selenite, selenate, or any other selenium compound is not available.

### Applicability to other GFAA spectrometers

The HPLC-GFAAS system described in this paper can be used for the separation and determination of compounds containing any element that can be detected with electrothermal AAS.

Other spectrometers that use an analogous concept of control can be coupled with a chromatographic system by adapting the interface accordingly. Compact instruments may cause difficulties because they usually offer no direct access to the electronics controlling the autosampler and the spectrometer.

The whole software, or parts of it, can be used and adapted for the chromatographic evaluation of signal

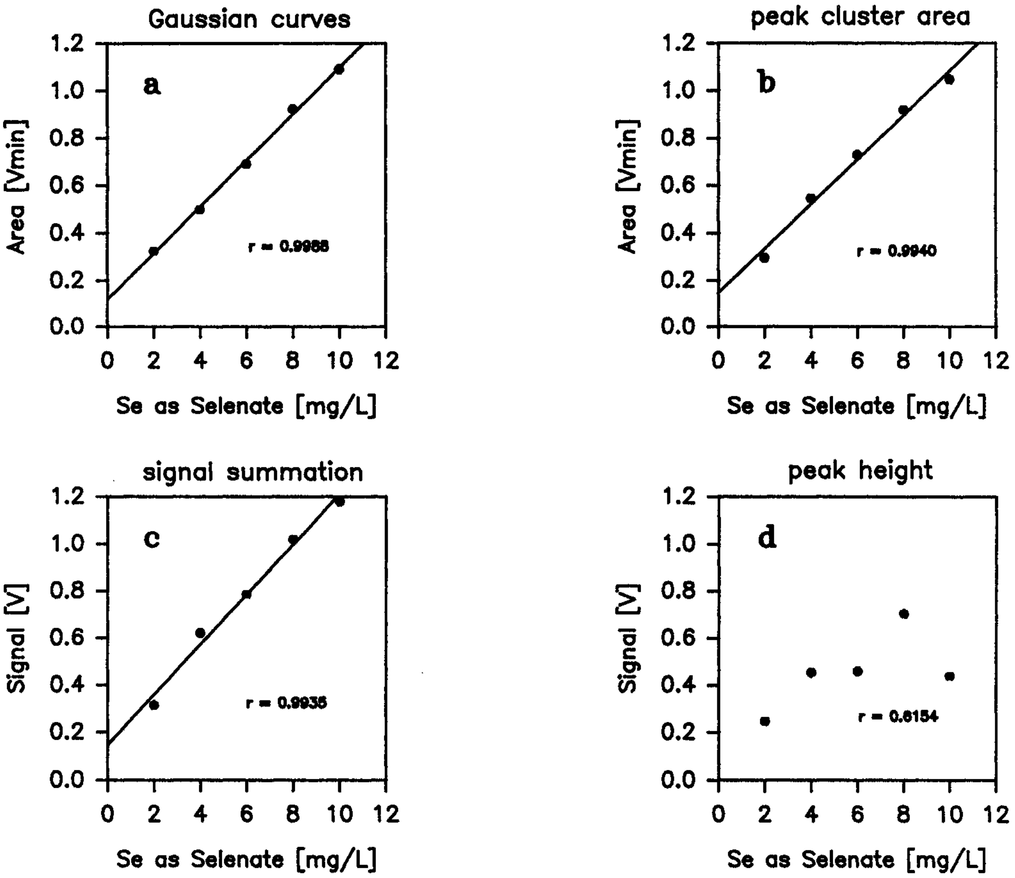

*Figure 7. Quantification of chromatographic peaks with different procedures: (a) Gaussian curves; (b) area of peak clusters with trapezium integration; (c) summation of signals within peak limits; (d) maximal signal within peak limits. Experimental parameters are the same as in figure 5, except for the sample volume (100 μl).*

responses recorded by other detectors; in this case, the data must be imported into the program in a proper format.

### References

1. Bernhard, M., Brinckman, F. E. and Irgolic, K. J., in *The Importance of Chemical 'Speciation' in Environmental Processes*, Ed. Bernhard, M., Brinckman, F. E. and Sadler, P. J. (Springer, Berlin-Heidelberg-New York, 1986), p. 7.
2. Cantillo, A. Y. and Segar, D. A., in *Proceedings of the International Conference on Heavy Metals in the Environment* (Toronto, Canada, 1976), p. 183.
3. Brinckman, F. E., Blair, W. R., Jewett, K. L. and Iverson, W. P., *Journal of Chromatographic Science*, **15** (1977), 493.
4. Vickrey, T. M., Buren, M. S. and Howell, H. E., *Analytical Letters*, **A11** (1978), 1075.
5. Vickrey, T. M., Howell, H. E. and Paradise, M. T., *Analytical Chemistry*, **51** (1979), 1880.
6. Stockton, R. A. and Irgolic, K. J., *International Journal of Environmental Analytical Chemistry*, **6** (1979), 313.
7. Vickrey, T. M. and Eue, W., *Journal of Automatic Chemistry*, **1** (1979), 198.
8. Fish, R. H., Komlenic, J. J. and Wines, B. K., *Analytical Chemistry*, **56** (1984), 2452.
9. Haswell, S. J., Stockton, R. A., Bancroft, K. C. C., O'Neill, P., Rahman, A. and Irgolic, K. J., *Journal of Automatic Chemistry*, **9** (1987), 6.
10. Pinel, R., Benabdallah, M. Z., Astruc, A., Potin-Gautier, M. and Astruc, M., *Analusis*, **12** (1984), 344.
11. Ebdon, L., Hill, S. and Ward, R. W., *Analyst*, **112** (1987), 1.
12. Service manual for the model 170-70 Zeeman effect atomic absorption spectrophotometer (Hitachi Ltd, Tokyo, Japan, 1977).
13. Press, W. H., Flannery, B. P., Teukolsky, S. A. and Vetterling, W. T., in *Numerical Recipes – The Art of Scientific Computing* (Cambridge University Press, Cambridge, 1989), p. 495.
14. Press, W. H., Flannery, B. P., Teukolsky, S. A. and Vetterling, W. T., in *Numerical Recipes – The Art of Scientific Computing* (Cambridge University Press, Cambridge, 1989), p. 86.
15. Brinckman, F. E., Jewett, K. L., Iverson, W. P., Irgolic, K. J., Ehrhardt, K. C. and Stockton, R. A., *Journal of Chromatography*, **191** (1980), 31.

